# Playing It Safe: Assessing Cumulative Impact and Social Vulnerability through an Environmental Justice Screening Method in the South Coast Air Basin, California

**DOI:** 10.3390/ijerph8051441

**Published:** 2011-05-06

**Authors:** James L. Sadd, Manuel Pastor, Rachel Morello-Frosch, Justin Scoggins, Bill Jesdale

**Affiliations:** 1 Department of Environmental Science, Occidental College, Los Angeles, CA 94001, USA; E-Mail: jsadd@oxy.edu; 2 Program on Environmental and Regional Equity, University of Southern California, Los Angeles, CA 90089, USA; E-Mails: mpastor@college.usc.edu (M.P.); scogginj@college.usc.edu (J.S.); 3 Department of Environmental Science, Policy and Management, University of California at Berkeley, Berkeley, CA 94720, USA; E-Mail: bill.jesdale@gmail.com (B.J.); 4 School of Public Health, University of California at Berkeley, Berkeley, CA 94702, USA

**Keywords:** environmental justice, environmental health, geographic information systems, social vulnerability, cumulative impacts

## Abstract

Regulatory agencies, including the U.S. Environmental Protection Agency (US EPA) and state authorities like the California Air Resources Board (CARB), have sought to address the concerns of environmental justice (EJ) advocates who argue that chemical-by-chemical and source-specific assessments of potential health risks of environmental hazards do not reflect the multiple environmental and social stressors faced by vulnerable communities. We propose an Environmental Justice Screening Method (EJSM) as a relatively simple, flexible and transparent way to examine the relative rank of cumulative impacts and social vulnerability within metropolitan regions and determine environmental justice areas based on more than simply the demographics of income and race. We specifically organize 23 indicator metrics into three categories: (1) hazard proximity and land use; (2) air pollution exposure and estimated health risk; and (3) social and health vulnerability. For hazard proximity, the EJSM uses GIS analysis to create a base map by intersecting land use data with census block polygons, and calculates hazard proximity measures based on locations within various buffer distances. These proximity metrics are then summarized to the census tract level where they are combined with tract centroid-based estimates of pollution exposure and health risk and socio-economic status (SES) measures. The result is a cumulative impacts (CI) score for ranking neighborhoods within regions that can inform diverse stakeholders seeking to identify local areas that might need targeted regulatory strategies to address environmental justice concerns.

## 1. Introduction

Air pollution has long been recognized as a high priority for both environmental health and justice by researchers, government regulators, and community residents [1–4] In California in particular, there is consistent evidence indicating patterns of both disproportionate exposure to air pollution and associated health risks among minority and lower-income communities [5–9]. These same communities also face challenges associated with low social and economic status, including psychosocial stressors, which make it more difficult to cope with exposures and may be connected with the persistence of environmental health disparities [10–12].

Environmental justice (EJ) advocates have argued that scientists and regulatory agencies should better account for the cumulative impacts (CI) of environmental and social stressors in their decision-making and regulatory enforcement activities [13,14]. These advocates and others have suggested that traditional chemical-by-chemical and source-specific assessments of potential health risks of environmental hazards do not reflect the multiple environmental and social stressors faced by vulnerable communities, which can act additively or synergistically to harm health [15–17]. Regulatory agencies are beginning to respond to the National Research Council’s call for the development “cumulative risk frameworks” within their scientific programs and enforcement activities [18]. In California, the Office of Environmental Health Hazard Assessment maintains a Cumulative Impacts and Precautionary Approaches Work Group which has advised the Agency in its efforts to develop guidelines for consideration of cumulative impacts within the different programs of the California Environmental Protection Agency [19].

This approach represents an advance from earlier definitions of environmental justice concerns which emphasized the racial/ethnic make-up or income levels of the communities in question (such as President Clinton’s Executive Order #12898 which directed federal agencies to focus on “minority communities and low-income communities”). Still, the work to develop more sophisticated tools for assessing cumulative impacts and environmental disparities is in its infancy. For example, Su and colleagues developed an index to characterize inequities by race/ethnicity and SES in the cumulative impacts of environmental hazards at the regional level, which allows for comparisons at large geographic scales [20]. However, this approach is not conducive to ranking and assessing distributional patterns of CI at more local, neighborhood-level scales within regions, which has been a primary concern for EJ advocates and some regional air quality agencies. These within-region CI assessments are important because industrial clusters, as well as land-use planning decisions, are often rooted within metropolitan regions; thus regulatory interventions to mitigate the cumulative impact of environmental and social stressors often require regionally-specific strategies [21,22].

The U.S. EPA has also been developing a GIS-based cumulative impacts screening tool, known as the Environmental Justice Strategic Enforcement Assessment Tool (EJSEAT) [23] to identify areas with disproportionately high and adverse environmental health burdens nationwide. EJSEAT defines a set of 18 cumulative impacts indicator metrics organized into four categories (demographic, environmental, compliance, and health impact), scales these values within each state (rather than, say, the metropolitan region or the air basin) and then applies to each census tract a composite score. However, EJSEAT is considered to be a “draft tool in development, currently under review and intended for internal EPA use only” and it has certain limitations due to the requirement for national consistency. These limitations include the fact that much of the non-Census data used to develop indicators is limited to that generated by EPA itself and sources of EJ concern, such as land use activity, are not captured. Additionally, county level health impacts information is imputed to census tracts, thus, ignoring much of the important variation by neighborhood. Compliance data, which consists of inspections, violations, formal actions and facility density, is problematic; for example, more inspections could indicate better regulatory oversight or worse behavior on the part of facilities. Moreover, violations and actions are not ranked by severity, leading one assessment to suggest that “the application of compliance statistics are so uncertain in meaning that their use as an indicator is highly questionable” [24].

We present an Environmental Justice Screening Method (EJSM) that facilitates examination of patterns of cumulative impacts from environmental and social stressors across neighborhoods within regions. We demonstrate an application of the EJSM to the six county area covered by the Southern California Association of Governments (SCAG), a region that is home to nearly half (48.8%) of California’s population. We specifically sought to create an EJSM that relied on publicly available data in order to facilitate its application to different contexts, as well as the addition of new data layers and the updating of information as needed.

The analytical work to develop the EJSM was solicited and funded by the California Air Resources Board (CARB). Therefore, the method was developed with considerable input from Agency scientists as well as an external scientific peer review committee that provided ongoing advice on methods and metrics selection. We also solicited feedback from environmental health and environmental justice advocates regarding appropriate metrics and we previewed preliminary results for their feedback. This strategy of soliciting peer review from agency personnel, scientific colleagues and community stakeholders was aimed at ensuring that the final EJSM was methodologically sound and transparent to diverse audiences in the regulatory, policy and advocacy arenas. As discussed below, the multiple audiences also required certain trade-offs; in particular, we made several choices to insure that the method would be more easily understood by community stakeholders as that would encourage their acceptance of the EJSM as a reasonable approach for regulatory guidance.

## 2. Experimental Section

### 2.1. Methods

The EJSM allows a mapping of cumulative impacts using a set of 23 health, environmental and social vulnerability measures organized along three categories: (1) hazard proximity and land use; (2) estimated air pollution exposure and health risk; (3) social and health vulnerability. Individual indicators and data sources are summarized in Table 1.

The EJSM involves a four-step process: (a) an initial GIS spatial assessment to create a detailed regional base map for estimating hazard proximity; (b) the use of GIS techniques to appropriatly summarize the resulting hazard proximity indicators for each of the region’s census tracts; (c) the coupling of the resulting tract level scores with tract level data on air pollution exposure and/or health risk as well as data on social and health vulnerability, (d) a cumulative ranking based on all the tract-level indicators that is then presented visually.

**Figure 1.**
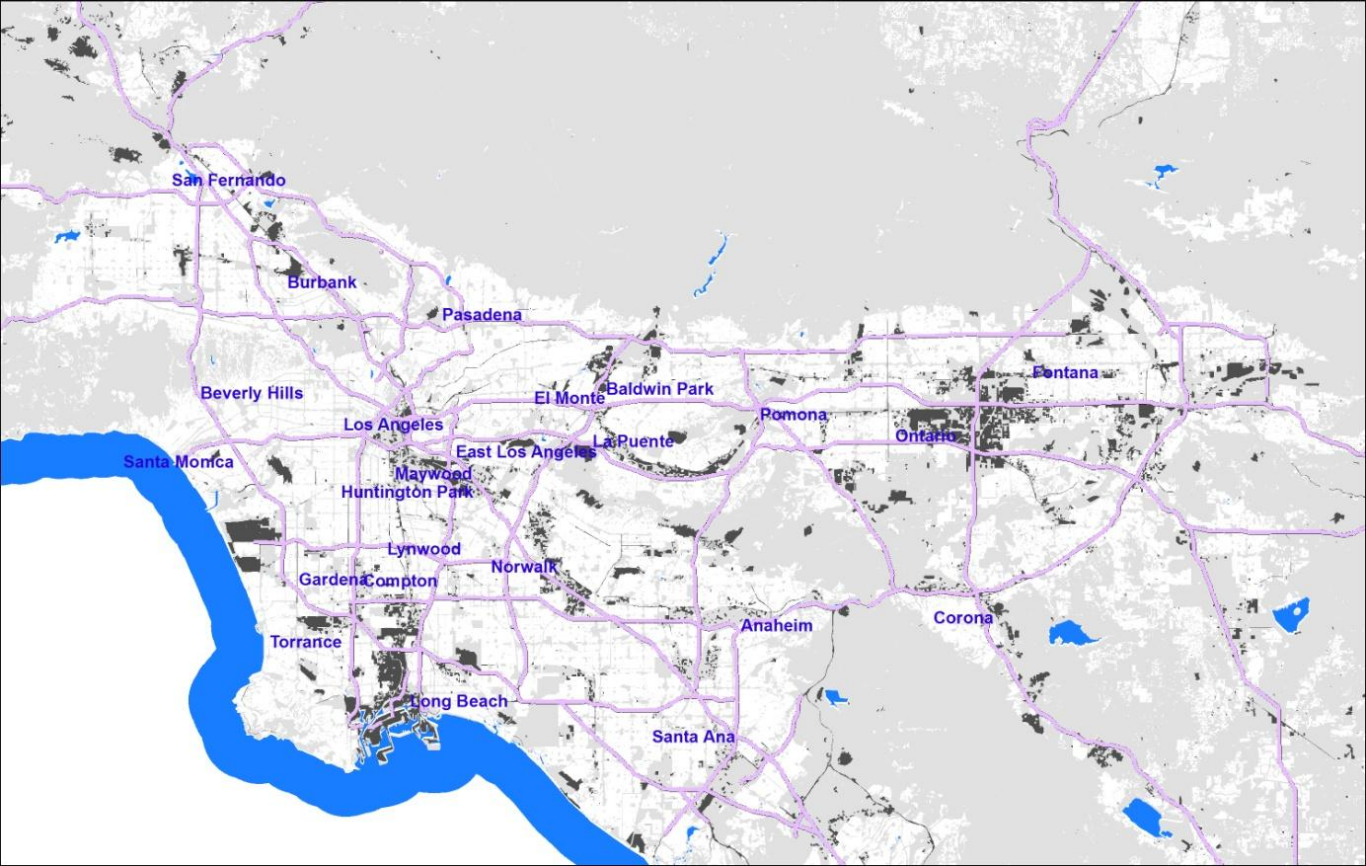
Map of a portion of the study area showing CI Polygons in white, and areas not scored (including open space, vacant land, industrial land use, *etc.*) in gray.

The regional base map is constructed by integrating specified residential and sensitive land use classes (see below) as classified by the California Air Resources Board [25]. This focuses CI screening on areas with land uses where people reside or locations hosting schools, hospitals, day care centers, parks and other sensitive receptor locations. Areas that are, for example, strictly industrial or commercial or undeveloped open space are not included in the regional base map (see Figure 1).

To geographically link the regional base map with the tract-level metrics of social/health vulnerability and air pollutant exposure/health risk, the residential and sensitive land use polygons were intersected using a GIS procedure with census block polygons from the 2000 Census, to create a base map composed of neighborhood-sized cumulative impact (CI) polygons, each with a known land use class and attribute key to attach census information. The base map for the Southern California area we developed consists of over 320,000 CI polygons, with the median area of these polygons being 0.017 square kilometers. There are slightly less than 145,000 populated census blocks in the same area, suggesting that our base units are generally portions of blocks.

### 2.2. Data and Scoring

The regional base map and the buffer-based hazard proximity scoring were derived using GIS. We also used Statistical Analysis Software (SAS) 9.2 and Statistical Package for the Social Sciences (SPSS) 17.0 for distributional calculations and tract-level scoring to facilitate documentation and error-checking.

The first step in our analysis involved attaching to each of the CI polygons on our regional base map a set of hazard proximity indicators and then summarizing these to create scores at the tract level. We then attached the other metric categories (air pollution exposure and health risk; and social and health vulnerability) and calculated a total CI score. Examining each metric category separately and then combining them into a total score facilitates screening for relative cumulative impacts of environmental and social stressors between neighborhoods in a structured manner that can inform regulatory decision-making in diverse regulatory and community contexts [26].

#### 2.2.1. Hazard Proximity and Land Use Indicators

This category captures the location of stationary emission sources and sensitive land uses based on the California Air Resources Board (CARB) Air Quality and Land Use Handbook which recommends buffer distances to separate residential and other sensitive land uses from potential hazards in order to protect susceptible populations.[25] Susceptible populations are considered to be young children, pregnant women, the elderly, and those with existing respiratory disease, who are especially vulnerable to the adverse health effects of air pollution [27]. The non-residential sensitive land uses indicated by CARB include schools, childcare centers, urban playgrounds and parks, and health care facilities, and senior residential facilities.

Residential and sensitive land use features were mapped using several data sources, including regional land use spatial data from the Southern California Association of Governments (SCAG) [28], state regulatory agency databases, and geocoded locations from address lists. The residential uses were straightforward as housing is clearly delineated in the SCAG 2005 land use data layer. That layer also had several of the non-residential sensitive uses. However, not all sensitive land uses are available as polygon features in this data layer, due to limitations either of the spatial resolution or other issues. For example, some commercial and other facilities contain childcare centers or health care facilities that are not mapped separately. In addition, because of a recent boom in school construction in California, some schools post-date the vintage of the SCAG land use layer.

To address this shortcoming, point locations for these additional sensitive land use features were identified from other data sources, and address geocoding was used to create point feature spatial layers. School location points, for example, were automated using the address list provided by the California Department of Education (2005); public and private schools were included. Childcare centers were automated from the addresses provided from a search of Standard Industrial Code (SIC) 8350 and 8351 using the D&B (formerly Dunn and Bradstreet) Business Information Service; senior housing facilities were similarly automated (SIC 8361). Point locations of healthcare facilities were obtained from the California Spatial Information Library (http://www.atlas.ca.gov/download.html). To avoid duplication with polygon features, any point feature that intersected an equivalent polygon feature was dropped—for example, a point location for a school that is located within a SCAG land use school polygon was deleted.

Finally, because representing these features as dimensionless points would result in misclassification of proximity metrics, we assigned a minimum area to each point feature by creating circular buffers. The size of these buffers was selected based upon the area of the smallest equivalent land use in the SCAG Land Use data layer, with the rationale being that the smallest SCAG polygons represent the limit of the spatial resolution of the SCAG data, and smaller features were simply not mapped.

We then added to the map point source locations prioritized by CARB as significant sources of air pollution and also prioritized in community scoping sessions as locations of concern. Point feature locations include: (a) facilities from the Community Health Air Pollution Information System (CHAPIS)—a subset of the California emissions inventory with criteria and air toxics emissions of primary concern for health impacts [29]; (b) chrome-plating facilities identified from the California air toxics emissions inventory [30]; and (c) selected hazardous waste facilities from the California Department of Toxic Substances Control (DTSC) [31]. Stationary emission sources prioritized by CARB (CARB 2005) include rail facilities, airports, intermodal distribution facilities, refineries and ports where diesel emissions are concentrated; these are added as polygon and/or line features from the land use layer.

Each CI polygon—consisting of either a residential or sensitive land use—was scored as follows. We first constructed buffers at 1,000 feet, 2,000 feet, and 3,000 feet (*ca*. 305, 610 and 915 m, respectively) from the boundary of each polygon. The 1,000 foot distance was chosen because it is the standard that CARB generally applies in its community health risk assessments and is specified in its land use manual [25]; we also included hazards within two other bands (1,000–2,000 feet and 2,000–3,000 feet) because there is some degree of locational inaccuracy in the GIS data making strict buffering problematic, and some features (e.g., geocoded stationary hazards) may be spatially represented as point features just outside a buffer but, in reality, are polygons that stretch across buffers.

**Figure 2.**
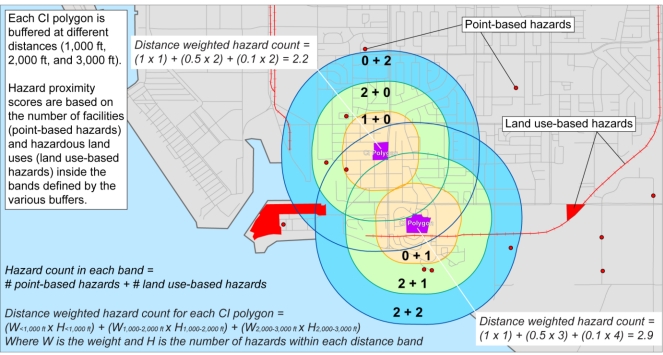
Method for assessing hazard proximity for CI polygons.

The number and type of sources within each of these buffer distances was determined for every CI polygon; a similar procedure is done for all hazards represented as area features (e.g., airports, refineries, railroad tracks). We then utilized a distance-weighted scoring procedure where the influence of the hazards on the sum attached to the CI polygon diminishes with distance (Figure 2) as those places with proximity to numerous air quality hazards are assumed to be more highly impacted. We applied this tiered buffering approach rather than a continuous distance-weighting method to ensure that the hazard and land use scoring was transparent to community stakeholders. Using this method, the summed point totals for each CI Polygon in the Southern California area we examined ranges from 0 to 9.8.

We then added to the distance- weighted hazard proximity counts a binary dummy variable indicating whether the CI Polygon was residential land (0) or a non-residential sensitive land use. A tract-level hazard proximity score is then calculated based on the hazard proximity and sensitive land use measure by attaching to each CI polygon a population weight derived from assigning population using the underlying intersection of census block data and polygon land area; we then used that value to weight the scores to a census tract average score for hazard proximity/sensitive land use. The downside of this strategy is that it can underweight the hazard proximity measure if a block that is attached to a particular polygon has either no residents or a low population (for example if part of the block is a school). An alternative approach involves area weighting; however, this approach can overweight larger CI polygons which may have few residents. As the results were generally similar and our focus was on community impacts, we conducted population-weighting.

Finally, a quintile ranking from 1 (low) to 5 (high) was applied to derive a tract-level score which integrates the presence of both sensitive and hazardous land uses. More complex ranking strategies were available, including the utilization of Jenks’ natural breaks for these figures or the determination of a mean and standard deviation, with four breaks determined as being more than one standard deviation above (or below) the mean or between one standard deviation and the mean. However, quintile ranking yielded results similar to the more complex approaches and were more transparent to community stakeholders; this was also the case for the other variables discussed below.

#### 2.2.2. Health Risk and Exposure Indicators

This category includes five metrics of air pollution concentration estimates or health risk estimates associated with modeled air toxics exposures, all calculated at the census tract level. They include toxicity weighted hazard scores for air pollutant emissions from the 2005 Toxic Release Inventory facilities included in the U.S. EPA’s Risk Screening Environmental Indicators, estimated at the census tract level using a Gaussian-plume fate-and-transport model (RSEI-Geographic Microdata database) [32,33]; the CARB cumulative estimated lifetime cancer risk associated with ambient air toxics exposures from mobile and stationary sources for 2001 [34,35]; tract-level estimates of cumulative respiratory hazard derived from the 1999 National Air Toxics Assessment (NATA) [36]; tract-level ambient concentration estimates interpolated from the CARB statewide criteria air pollutant monitoring network for PM_2.5_ and ozone concentration estimates and averaged for 2004–2006 [34].

Intermediate scores for each health risk and exposure metric were calculated based on quintile distribution rankings (with scores ranging from 1–5) for all tracts in the study area. As these health risk and exposure metrics are at the tract level, each CI polygon receives the metric score for its host census tract and the ranking is done at the tract level. For example, a CI polygon located in a tract that ranks in the least impacted 20% for each of the five exposure and health risk metrics (PM_2.5_ concentration, ozone concentration, estimated cumulative cancer risk for air toxics, estimated respiratory hazard for air toxics, and toxicity-weighted pollutant emissions from RSEI) would receive a total health risk and exposure score of 5 (5 metric scores of 1), whereas a tract that ranked in the highest quintile for all five metrics would have a total exposure and health risk score of 25 (5 metric scores of 5). These total intermediate scores are then re-ranked into quintiles by tract to derive the final score for this air pollution exposure/health risk category, which ranges from 1 to 5.

#### 2.2.3. Social and Health Vulnerability Indicators

This category of indicators includes tract level metrics identified by the social epidemiology and environmental justice research literature as important factors for adverse health outcomes and statistically significant determinants of patterns of disparate impact. Variables from the 2000 U.S. Census [37] include measures of race/ethnicity (% residents of color), poverty (% residents living below twice national poverty level), wealth (% home ownership using % living in rented households), educational attainment (% population over age 24 with less than high school education), age (% under 5 years old and % over 60 years old), and linguistic isolation (% residents above the age of 4 in households where no one over age 15 speaks English well). Non-census metrics include % voter turnout (% votes cast among all registered voters in the 2000 general election) [38] as a proxy for degree of engagement in local decision-making (which has been linked to community health status [39]), and adverse birth outcomes (% preterm or small for gestational age infants 1996–03) both of which are sensitive health endpoints that reflect underlying community health status (California Automated Vital Statistics System, 2006, unpublished data).

Intermediate social and health vulnerability indicator scores were calculated using the same quintile distribution and normalization technique employed for the health risk and exposure indicators, above, with scores ranging from 1 to 5. To ensure that social and health vulnerability scores were not distorted by missing data or based upon anomalously small populations, tracts with fewer than 50 people and those with fewer than six indicator values were not scored (n = 34 out of 3,381 tracts or about 1% of census tracts). Some of these tracts had already been eliminated in the hazard proximity scoring phase owing to having no residential land. To insure comparability between tracts with all metrics and those tracts missing 1 to 4 metrics, we summarized the ranks in the individual metrics but then calculated a score based on dividing that sum by the number of non-missing metrics.

## 3. Results and Discussion

**Figure 3.**
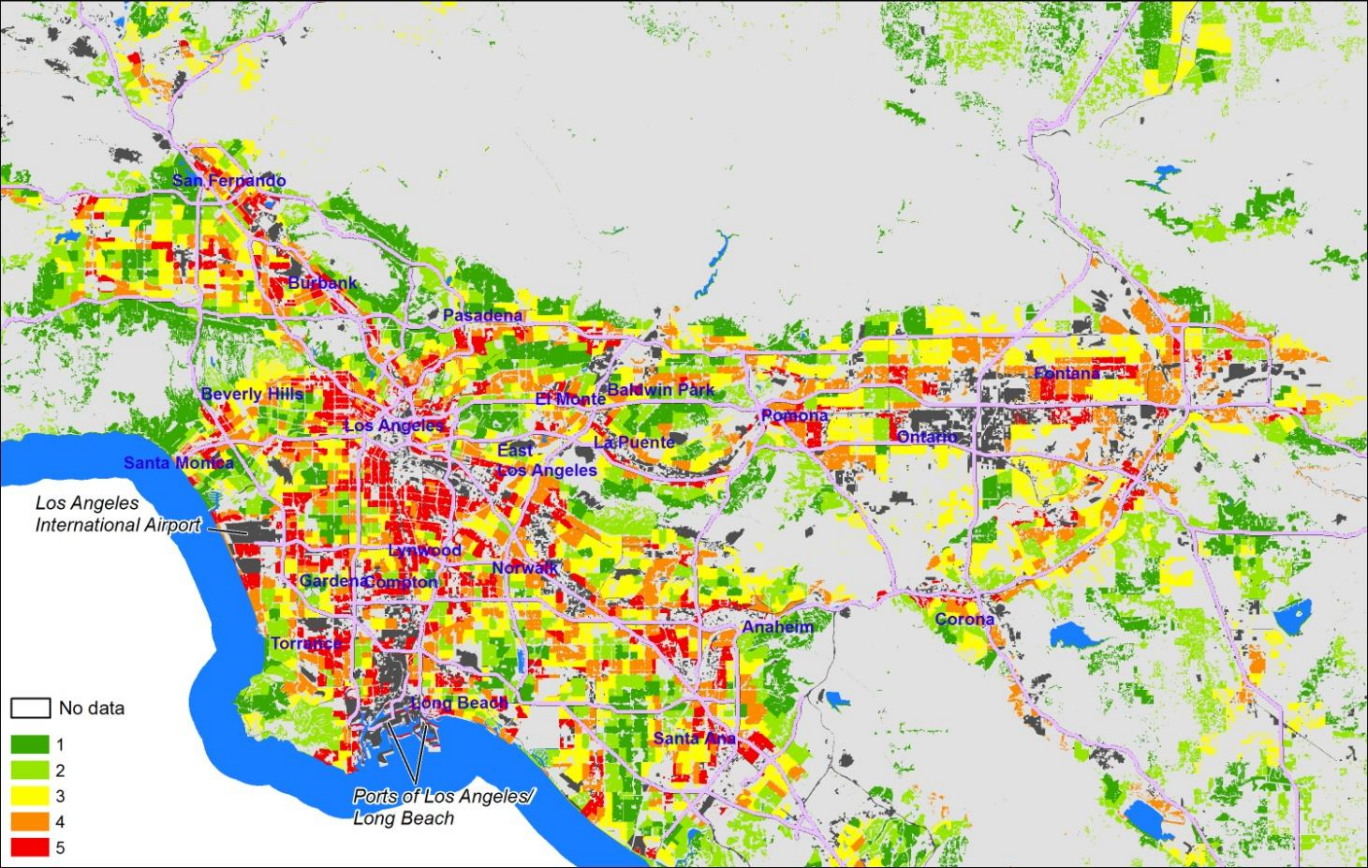
Hazard proximity and sensitive land use quintile scores at the tract level (mapped on CI polygons)—South Coast Air Quality Management District (SCAQMD), California.

Mapping the intermediate EJSM scores for the three indicator categories at the census tract level reveals some interesting geographic patterns. The maps shown below cover only the South Coast Air Quality Management District (SCAQMD) portion of the Southern California region studied, as most of the variation in scores is represented in this area. Areas with high hazard proximity and sensitive land use scores (Figure 3) tend to correspond with the more densely populated areas, and either tend to cluster around major industrial centers or follow major transportation corridors. High scores are typical in areas with populations characterized by high minority, low income populations, and adjacent to sectors of concentrated industrial activity (shown in dark gray), such as the Ports of Los Angeles/Long Beach, the Los Angeles International Airport, and the industrial core of Los Angeles running from the ports to downtown L.A.

**Figure 4.**
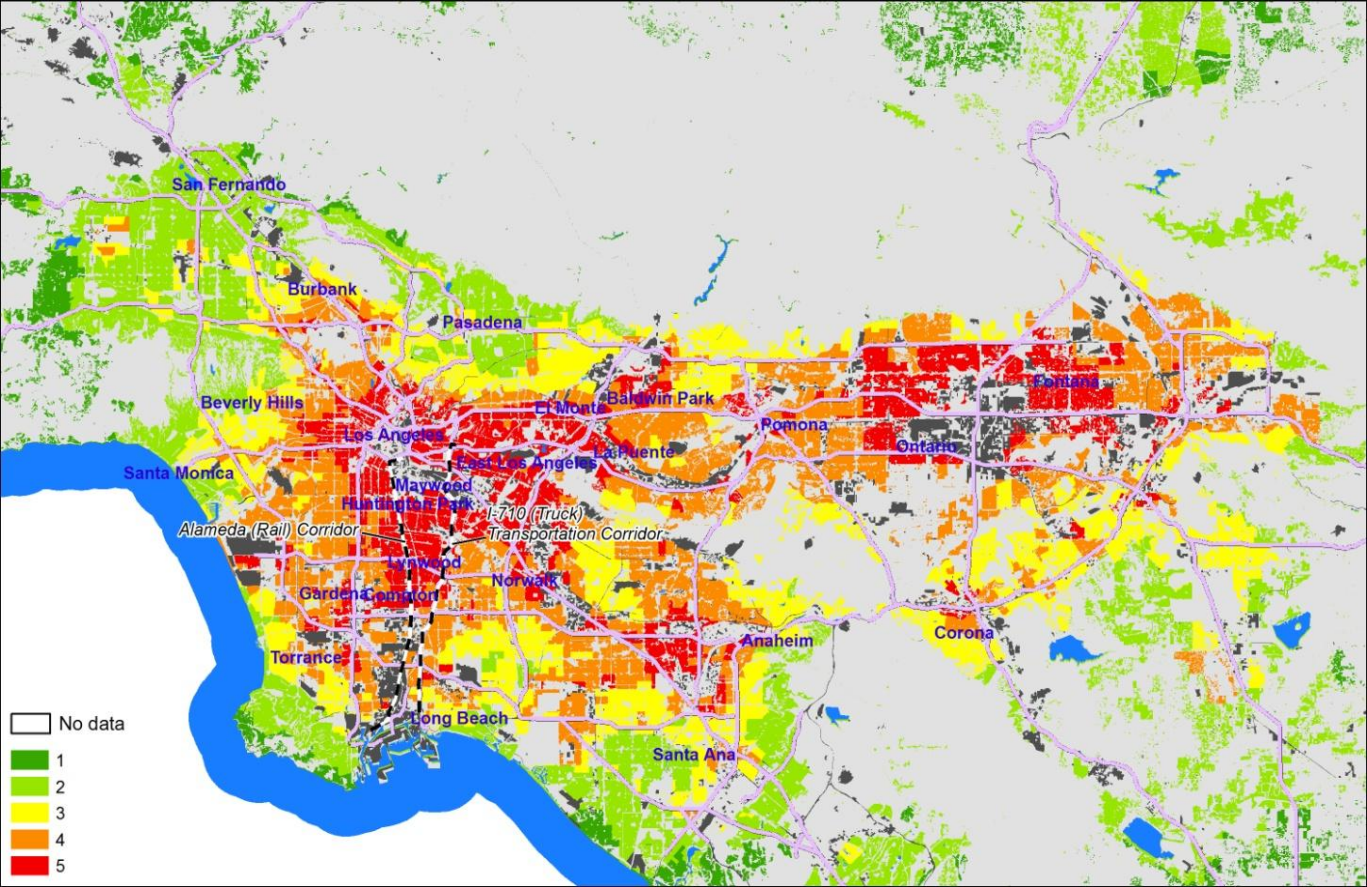
Air pollution exposure and health risk quintile scores at the tract level (mapped on CI polygons)—SCAQMD.

The geographic distribution of the Health Risk and Exposure scores (Figure 4) is less complex, but with a clear concentric pattern with little fine-scale variation with broad areas with a single score. Areas with the highest scores surround heavily industrialized areas, including central and East Los Angeles, the Alameda corridor connecting downtown to the ports along the 710 transportation (truck, rail, freeway) corridor, and the industrial centers in Baldwin Park and east of Ontario International Airport. Coastal and foothill neighborhoods are characterized by low scores, and the apparent effects of the freeway system on the overall pattern are minor. This pattern is similar to the results of the MATES III (Multiple Air Toxics Exposure Study) project which evaluated and mapped health risks associated with air toxics and diesel particulates using the SCAQMD emissions inventory and monitoring programs [40] even though the MATES analysis is done at a much coarser level of spatial resolution, and includes mapping across all land use types. This suggests that this metric category of the EJSM is consistent with other screening approaches; the innovation here is combining this with other dimensions as well as the adoption of a more transparent and community-engaged approach to developing the EJSM.

**Figure 5.**
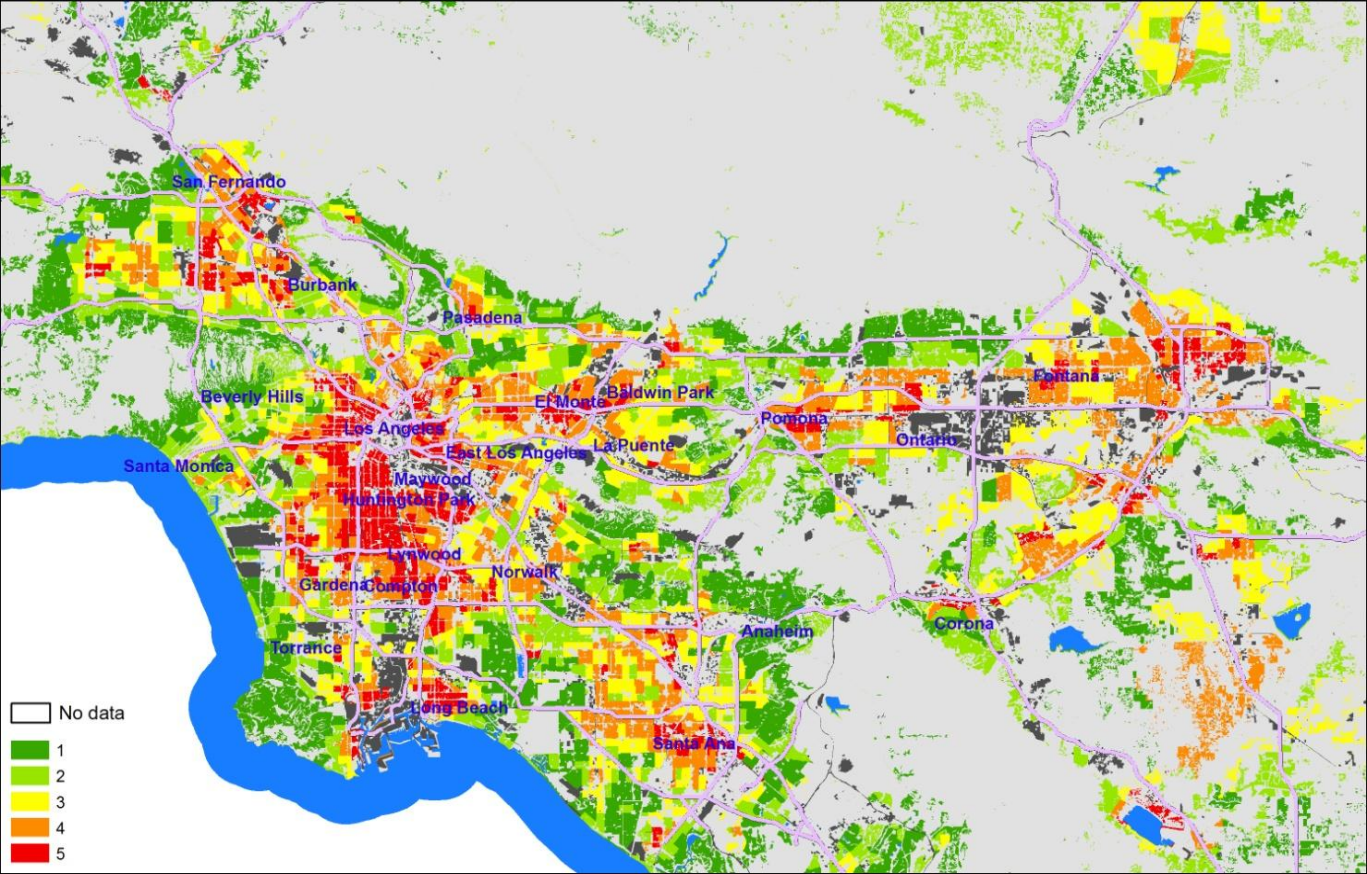
Social and health vulnerability quintile scores at the tract level (mapped on CI polygons)—SCAQMD.

Social and Health Vulnerability scores (Figure 5) reflect the well documented pattern of residential segregation in metropolitan Los Angeles by SES variables of race and class. Many of the same neighborhoods bearing the burden of high exposure to air pollution and its attendant health risks are also those where the most vulnerable populations are also concentrated.]

**Figure 6.**
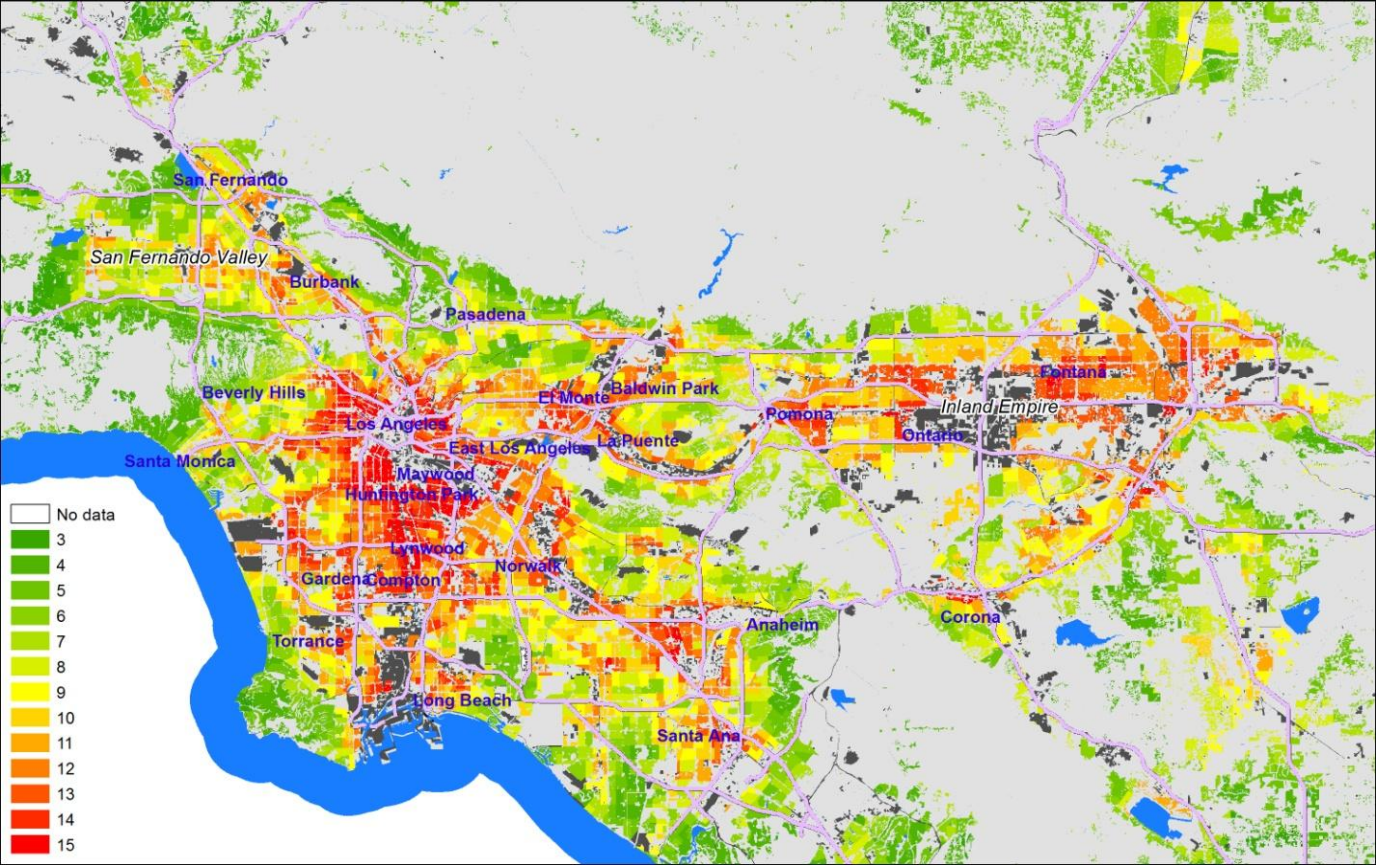
Total cumulative impact quintile scores at the tract level (mapped on CI polygons)—SCAQMD.

The three intermediate category scores are summed into a Total Cumulative Impacts (CI) Score that ranges from 3–15 (Figure 6). For visual representation, these scores are attached in the GIS system to each CI polygon (since that focuses attention on the residential and sensitive land use areas) but they are based on tract-level scores. It is worth noting that the regional distribution of Total CI Scores is near normal.

Certain areas, like communities near the ports and airports as well as the heavily impacted Pacoima neighborhood in the San Fernando Valley have the highest CI scores (shown in red). Community activism around environmental justice has occurred in these areas and they are often receiving targeted attention from regulators and policy makers. What is perhaps more useful is that the CI map also points to communities that do not have a record of organizing and have not brought themselves to the attention of regulators or decision-makers, such as East Los Angeles (which is intersected with freeways and populated with smaller hazard), Pomona east of Los Angeles, and parts of the Inland Valley (Riverside and San Bernardino Counties). From the view of regulators, the map helps direct attention to places where specific attention may be needed to address environmental health concerns not usually considered; from the point of view of community stakeholders, the map highlights locations where residents may need to be educated and engaged to address environmental hazards.

A number of science-policy choices must be made during the development of any screening method and the EJSM is no exception. For example, we chose to include hazard proximity (and sensitive land use designation) as well as air quality and health risk measures. While it can be argued that the health risk measures are most important and that including a category for hazard proximity is duplicative, we believe that CI screening should include metrics that are also meaningful for land-use and planning contexts to better account for the larger impact of place on community health. Indeed, studies indicate that communities living near industrial and hazardous waste sites experience an increased risk of psychosocial stress and mental health impacts in addition to other health outcomes [41,42]. Therefore, in order to be accessible to a variety of community, agency and other regulatory stakeholders, we chose not to limit the EJSM to quantitative risk estimates of potential health impacts.

We also did not to attach explicit weights to any of the three metric categories or to any of the specific metrics within each category (e.g., rankings for the cumulative estimated lifetime cancer risk associated with ambient air toxics and ranking for the tract-level ambient PM_2.5_ concentration estimates both have the same weight within our category of air pollution-related estimated health risk). Our decision was based on the fact that there is a paucity of scientific evidence that provides specific guidance for a particular weighting scheme and it was also guided by community stakeholder feedback expressing worries about arbitrary weights. We note, however, that the EJSM has been developed with enough flexibility to allow for weighting of metrics if a specific decision-making context warrants such an approach. Weights could be assigned directly to metric scores, or the range of scores for specific metric categories could differ based on determinations of the strength of the data available. This latter approach is one that is currently being considered by California’s Office of Environmental Health Hazard Assessment [43].

Similarly, our use of quintiles as the basis to score metrics and to derive a single CI score was driven at least partly by our desire to have our method be more transparent and accessible to diverse audiences. As noted earlier, alternative approaches could use means and standard deviations to capture outlier CI tracts; however, since the health risk metrics are not normally distributed, this requires taking the mean and standard deviations of a logged measure. Since the relative ranking of tracts is not changed significantly by this more complicated procedure compared to quintile-based scoring, we chose the approach that is more accessible and more easily understood by the public. This is particularly important in policy areas like environmental justice where a pattern of distrust between agencies and community stakeholders might argue that simple and straightforward is best, at least in the initial phases of developing screening approaches.

We also note that the hazard proximity and land use dimension could be evaluated using different distance buffers than the ones we applied. We made use of CARB-specified land use buffers [25] but expanded the distance with multiple buffers and distance-weighting to account for potential locational inaccuracies of point and area emission sources. We also chose to summarize hazard proximity/land use scores to the tract level to harmonize the data from this category with the tract-level data from the air pollution exposure/health risk and social/health vulnerability categories. An alternative approach would have been to attach to each hazard proximity/land use polygon the tract-level exposure/health risk and social vulnerability scores. However, as we have suggested, this approach misrepresents the geographic accuracy of the health risk/exposure and social/health vulnerability metrics, all of which are calculated at the tract level. The tract level approach likely has the effect of lowering scores for those CI Polygons that are within the high range of the distribution because of the averaging at the tract level, possibly under-representing cumulative impacts for some neighborhoods.

## 4. Conclusions

The EJSM was developed as an approach for assessing patterns of cumulative impacts from environmental and social stressors across neighborhoods within regions, using Southern California as a case study. Relying on secondary data sources, the EJSM integrates and scores multiple metrics of environmental and social stressors to rank census tracts in a way that is rigorous yet transparent to diverse stakeholders, particularly regulators, policymakers and communities.

In part because we consider hazard proximity and land use to be an essential component of cumulative impact screening, we constructed the EJSM by intersecting a land use spatial layer with census block geography. This creates the distinct advantage of targeting CI screening in areas where people live or where there are sensitive receptors. However, this approach also poses one disadvantage, in that it relies on reasonably precise and well-classified land use data. This information is not uniformly available in all regions of California or elsewhere in the country.

Our future work will examine whether land use data with lower spatial resolution or different types of classification, such as automated classification of aerial photo and satellite imagery or land parcel data, might be utilized and how that would affect the accuracy of screening results. As the quality and availability of land use data continues to improve, we believe that this challenge is not likely to be a serious long-term liability for cumulative impacts screening methods such as the EJSM.

Of course, any screening method that assesses and compares cumulative impacts across diverse locations must be followed with further validation efforts to assess the accuracy of the data as well as the predictive value of the approach. Such validation work will require ground-truthing efforts to verify the locational accuracy in data sets and more refined air monitoring to assess whether and how interpolated exposure estimates are under- or over-predicting measured values in certain locations. Although discussion of this work is beyond the purview of this paper, we have begun to conduct such ground-truthing work in the Los Angeles area [44]. Finally, although the EJSM is flexible enough to allow for comparisons across different study areas (e.g., within regions or across the state) we have emphasized a regional application because generally land use planning, industrial and transportation development, and environmental regulation are regionally rooted and require regionally specific interventions to reduce hazard exposures or to address social and health vulnerability factors.

Despite these limitations, screening methods such as the EJSM can help regulators and policy makers more efficiently target their efforts to remediate cumulative impacts, environmental inequities, and focus regulatory action at the neighborhood level. Currently, the burden of proof is placed on communities to demonstrate the cumulative impacts of environmental and social stressors and push for action. CI screening such as the EJSM provides environmental policy and programs with a more proactive approach that removes this burden from vulnerable communities so that those without an active environmental justice movement or capacity for civic engagement can also receive regulatory attention and protection.

Moreover, the EJSM can advance regulatory decision-making and the implementation of environmental policies. In California, for example, recent climate change legislation, known as the Global Warming Solutions Act [45] mandates statewide goals to reduce greenhouse gas emissions and also requires consideration of how the law’s implementation will impact “communities that are already adversely affected by air pollution.” Moreover, the law requires that measures to reduce greenhouse gas emissions must be designed to “direct public and private investment toward the most disadvantaged communities in California and provide an opportunity for small businesses, schools, affordable housing associations, and other community institutions to participate in and benefit from statewide efforts to reduce greenhouse gas emissions.” As a result of this legislative mandate, CARB is developing its own EJ Screening approach, partly based on the EJSM, in order to comply with the law [46].

One key element of CI screening is the importance of soliciting stakeholder feedback on method development, metric choices and scoring approaches as these evolve. In addition to having extensive peer review by regulatory scientists and academic researchers, the EJSM was previewed multiple times by community stakeholders, including in early scoping sessions to solicit input on potential metrics. We also conducted some local “ground-truthing” exercises to test or verify the locational accuracy of secondary datasets [44,47].

Other regulatory agencies are currently grappling with the development of CI screening tools to inform decision-making in their regulatory programs. As noted earlier, US EPA has been developing an Environmental Justice Strategic Enforcement Screening Tool (EJSEAT) to identify communities experiencing disproportionate environmental and public health burdens for the purposes of enhancing enforcement and compliance activities [48]. Similarly, California’s Office of Environmental Health Hazard Assessment is also developing guidelines for cumulative impacts analysis to inform regulatory programs and enforcement activities within Cal-EPA [43]. The field of CI screening is likely to expand as land use and other data sources improve, and these efforts, if implemented, could be very helpful to identifying vulnerable communities and improving environmental health.

## Figures and Tables

**Table 1.** Summary of cumulative impact and vulnerability indicators used in the EJ Screening Method.

Sensitive land use indicators.		
**INDICATOR**	**GIS SPATIAL UNIT**	**SOURCE/DATE**
Childcare facilities	Land use polygons	Southern California Association of Governments (SCAG), 2005
	Buffered points	Dunn and Bradstreet by SIC code, 2006
Healthcare facilities	Land use polygons	SCAG 2005; California Spatial Information Library
Schools	Land use polygons	SCAG 2005
	Buffered points	CA Dept of Education 2005
Urban Playgrounds	Land use polygons	SCAG 2005
Environmental hazards and social vulnerability indicators.		
**INDICATOR**	**GIS SPATIAL UNIT**	**SOURCE/DATE**
**Hazardous Facilities and Land Uses**		
Air Quality Hazards		
Facilities in California Community Health Air Pollution Information System (CHAPIS)	Point locations	CA Air Resources Board (CARB) 2001
Chrome-platers	Point locations	CARB 2001
Hazardous Waste sites	Point Locations	CA Dept. Toxic Substances Control 2004
Hazardous Land Uses		
Railroad facilities	Land use polygons	SCAG 2005
	Line Features	National Transportation Atlas Database (NTAD)
Ports	Land use polygons	SCAG 2005
Airports	Land use polygons	SCAG 2005
	Line Features	NTAD 2001
Refineries	Land use polygons	SCAG 2005
Intermodal Distribution	Land use polygons	SCAG 2005
	Line Features	NTAD 2001

**INDICATOR**	**SOURCE/DATE**
**Health Risk and Exposure** all at census tract level	
Risk Screening Environmental Indicators (RSEI) toxic concentration hazard score	USEPA 2005
National Air Toxics Assessment respiratory hazard for air toxics from mobile and stationary emissions	USEPA 1999
Estimated cancer risks from modeled ambient air toxics concentrations from mobile and stationary emissions	CARB 2001
PM_2.5_ estimated concentration interpolated from CARB’s monitoring data	CARB 2004–06
Ozone estimated concentration interpolated from CARB’s monitoring data	CARB 2004–06
**Social and Health Vulnerability** all at census tract level	
% people of color (total pop–non-Hispanic white)	US Census 2000
% below twice the national poverty level	US Census 2000
Home Ownership–% living in rented households	US Census 2000
Housing Value–median house value	US Census 2000
Educational attainment–% >age 24 with <high school	US Census 2000
Age of residents–% <age 5	US Census 2000
Age of residents–% >age 60	US Census 2000
Linguistic isolation–% residents under age 4 in households where no one over age 15 speaks English well	US Census 2000
Voter turnout–% votes cast in general election	UC Berkeley Statewide Database 2000
Birth outcomes–% preterm and small for gestational age	CA Dept Public Health Natality Files 1996–2003
